# Motif V is an allosteric couple between the SARS-CoV-2 nsp13 nucleotide triphosphatase and helicase active sites

**DOI:** 10.1016/j.jbc.2026.111198

**Published:** 2026-01-23

**Authors:** Michael A. Mingroni, Brooke M. Enney, Lauren E. Malsick, Brian J. Geiss

**Affiliations:** 1 Department of Microbiology, Immunology and Pathology, Colorado State University, Fort Collins, Colorado, USA; 2 School of Biomedical and Chemical Engineering, Colorado State University, Fort Collins, Colorado, USA

**Keywords:** SARS-CoV-2, helicase, motif V, allostery

## Abstract

Non-structural protein 13 (nsp13), the Coronaviral RNA helicase, is an attractive antiviral target due to its importance in viral genome replication and highly conserved nature. Nsp13's processive dsRNA unwinding is driven by ATP hydrolysis in the nucleotide triphosphatase active site, which provides mechanical energy through conformational changes in the helicase domain to unidirectionally break hydrogen bonds between base pairs of bound dsRNA. Motif V is a conserved helical region between the nucleotide triphosphatase and helicase domains that has been previously shown to regulate ATP hydrolysis-mediated energy transduction within the flavivirus NS3 helicase. In this study, we characterized the role of the SARS-CoV-2 nsp13 Motif V in the regulation of ATPase-to-helicase crosstalk. Mutation of interacting Motif V residues T532 and S535 demonstrated increased rates of nucleic acid unwinding in an ATP-dependent manner, indicating the importance of the T532-S535 interaction in down-regulating energy transduction between the nucleotide triphosphatase and helicase domains. Furthermore, mutations to D534, which connects Motif V to the helicase domain through interaction with R560, severed the connection between the ATPase active site and the helicase domain *via* the disruption of a critical salt bridge. This connection was supported by the introduction of an L405D mutation, which attenuated helicase activity through repulsion of the D534 from R560. Overall, these data indicate that Motif V in SARS-CoV-2 nsp13 protein serves as a regulator of energy transduction in helicase function, similar to other families of positive-sense RNA viruses and helps define the mechanism of nsp13 helicase function.

Coronaviruses (Family: *Coronaviridae*), such as SARS-CoV-2, have caused significant global health crises due to their ability to rapidly spread and lead to acute respiratory illnesses. Coronaviruses primarily infect the respiratory system, and symptoms range from mild cold-like illness to life-threatening disease, including pneumonia, acute respiratory distress syndrome (ARDS), and multi-organ failure (1). The ability of Coronaviruses to mutate and adapt complicates containment efforts, as demonstrated by the rapid spread of SARS-CoV-2 and the emergence of new variants with heightened transmissibility (2). Betacoronavirus nonstructural proteins have high sequence conservation, and in particular, nsp13 possesses 99% sequence similarity across this genus, making it a valuable therapeutic target (3). Nsp13 in its dimeric form assembles with nsp7/8/12/14 to form the replication transcription complex (RTC), where nsp13 has been shown to participate in genome elongation in coordination with the RNA-dependent RNA polymerase and potential genome proofreading *via* backtracking RTCs during subgenomic transcription (4, 5, 6). The central role of nsp13 in the viral replication pathway makes the nsp13 helicase an attractive therapeutic target for the *Coronaviridae* family.

Nsp13 is translated as part of the larger orf1b polyprotein and is processed into its mature form by the 3CL^pro^ protease (7). Mature nsp13 colocalizes with the other nsps involved in the RTC in the perinuclear space within 4 h post infection, with dsRNA being detected within 2 h of infection (8). Cryo-EM structures of the RTC show two nsp13 molecules bound to the complex, one of which binds ssRNA leaving from the RdRp active site and entering the binding channel in the 1A domain and exiting the 1 B/2A in the 5′ to 3′ direction. The second nsp13 molecule makes a crucial interaction with the 1B domain of the first, presumably aiding in the conformational changes of the channel to allow RNA substrate to enter to drive enhanced helicase activity (9). Outside of its primary role of unwinding dsRNA, nsp13 has been shown to displace RNA-bound proteins, and the nsp13 NTPase domain is also capable of producing GDP from GTP that is used as a capping substrate for the NIRAN domain of nsp12 (10, 11). Additionally, nsp13 has been shown to help Coronaviruses evade the host antiviral response by inhibiting interferon (IFN) production through the degradation of TANK-binding kinase one and suppression of STAT1 signaling (12, 13).

Nsp13 belongs to the superfamily 1 (SF1) helicases and contains five characteristic domains, including two RecA-like domains (1A and 2A), a 1B domain, stalk domain and an N-terminal zinc-binding domain (Fig. 1*A*) (9). The superfamily designation for helicases are determined by the seven conserved motifs that make up the nucleotide binding sites, named Motif I, Ia, II, III, IV, V, and VI, found in the 1A and 2A domains, largely forming the NTP binding pocket and active site, and the RNA binding domain (14). Motifs I and II are the most robustly characterized across helicases, and have the residues involved in ATP phosphate/Mg^2+^ coordination, whereas Motifs Ia and IV interact with bound oligonucleotides (15, 16, 17). Motifs III, V, and VI have more variance between helicases, and form complex but critical interactions spanning both substrate binding sites bridging the two RecA domains (17, 18). Analysis of structural information in SARS-CoV-2 nsp13 shows Motif V as a 13 amino acid partially-helical motif which interacts with the phosphate backbone of bound RNA and extends into the ATP hydrolysis domain to make contact with the bound ATP phosphates (19, 20, 21). Motif V also is in a position to contact residues within both RecA-like subdomains 2A and 1A (Fig. 1*B*), providing a potential molecular communication channel between the enzyme subdomains involved in RNA interaction/unwinding and NTPase active sites.

**Figure 1:**
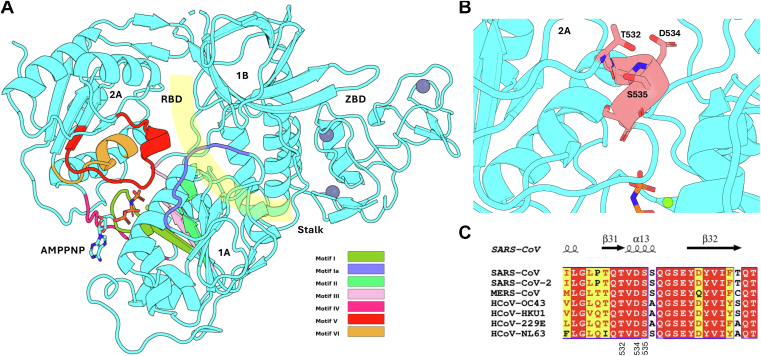
**Structure and conservation of Motif V in the SARS-CoV-2 nsp13 helicase**. *A*, structure of the SARS-CoV-2 nsp13 helicase based on PDB: 7NNO. Motif V in Domain 2A is colored in *red*, proximal to bound AMPPNP between Domains 2A and 1A, and the RNA binding domain is highlighted in *yellow* between Domains 1 A/2A and 1B. RBD = RNA Binding Domain, ZBD = Zinc Binding Domain. *B*, close-up view of the SARS-CoV-2 nsp13 Motif V region (*colored salmon*). Positions of residues ST532, D534 and S535 are noted. *C*, conservation of Motif V across the beta-coronavirus genus. Residues 532, 534, and 535 are noted.

Crystal structures of nsp13 (MERS-CoV, SARS-CoV and SARS-CoV-2) have provided significant insight into the static nsp13 structure, and biochemical analyses of nsp13 have shown the dynamic nature of the enzyme (19, 22, 23). The nsp13 helicase was first characterized in human coronavirus 229E to unwind double-stranded nucleotides unidirectionally from 5′ to 3′ in an ATP-dependent manner, driven by hydrolysis of ATP in the nearby phosphatase domain (24, 25). Within the NTPase, Motif I coordinates Mg^2+^ at a conserved lysine residue, which facilitates the non-discriminate binding and subsequent cleavage of the β- and γ-phosphates of NTPs and dNTPs, with a preference for purines (ATP and GTP) (26, 27). Activity of the NTPase is optimal when ATP is in molar excess to Mg^2+^, and mutational studies within the RNA binding pocket found NTPase activity is uncoupled from unwinding (10, 28).

To understand how NTPase activity drives the mechanics of the nsp13 helicase, molecular dynamics simulations have been used to explore allosteric effects within nsp13 induced by different ligand-bound states (29, 30). MD simulations are a powerful tool for predicting enzymatic behaviors and accelerating the exploratory process to assay development and biochemical analysis. Gaussian-accelerated Molecular dynamics (GaMD) has been used to analyze subtle conformational changes between four ssRNA-bound structures containing an empty NTPase site, ATP bound, ADP + P_i_, and ADP bound to mimic the process of ATP hydrolysis when RNA is bound (30, 31). Using this approach, they proposed an inchworm stepping translocation cycle for ATP-driven RNA unwinding by nsp13. The RNA binding pocket changed with different substrate-bound NTPase states, with Motif V driving the power stroke motion by shifting the proximity between neighboring phosphates to unidirectionally process RNA through the binding pocket. These simulation data corroborated earlier structural studies suggesting conformational differences between apo and ligand-bound states and highlighted allosteric pathways within nsp13 that function during ATP hydrolysis and RNA translocation (19). The connection between the ATPase active site and helicase domain in RNA helicases is crucial for the overall function of the enzyme, but has not been experimentally verified in the SARS-CoV-2 nsp13 helicase. Motif V couples energy transduction through structural motif stabilization was previously demonstrated for the flavivirus NS3 helicase by our group (32). Mutations at T407 and S411 in the West Nile virus NS3 helicase increased helicase activity, indicating that a hydrogen bond stabilizing the secondary structure of Motif V plays a role in negatively regulating the transfer of energy from the ATPase site to the helicase. The SARS-CoV-2 helicase shares a similar Motif V Thr-Ser pairing with NS3, and recent molecular dynamics simulations show Motif V residues having the closest contact to the RNA backbone, as well as the most movement between sampled conformations (29). Furthermore, Motif V interacted with modeled RNA backbone phosphates in sampled ATP hydrolysis states, suggesting that nsp13 Motif V was key in hydrolysis-driven RNA translocation *via* a power stroke motion (30).

Previously published crystal structures of nsp13 modeling the intermediates of ATP hydrolysis were used to analyze the α-helical secondary structure of Motif V (19). A side chain to backbone hydrogen bond is measured between both T532 and S535, both of which maintain close atomic distances not exceeding 3.1 Å in all intermediate states, demonstrating the strength of this motif. Taken with the kinetic observations in this study, the structural integrity of this loop is key in regulating energy transduction from the ATPase domain to the helicase binding cleft. The D534 is at its furthest from the R560 residue in the Apo state (Fig. 2*A*) and rotates to form contacts upon binding of ATP (Fig. 2*B*, represented by non-hydrolysable ATP analog, ADPNP). Finally, in the Pi-bound state representing a post-hydrolysis intermediate, the D534 has been pulled to form a salt bridge with R560 (Fig. 2*C*). L405 is situated between this interaction; thus, the mutation to an aspartic acid is proposed to disrupt this dynamic (30).

**Figure 2:**
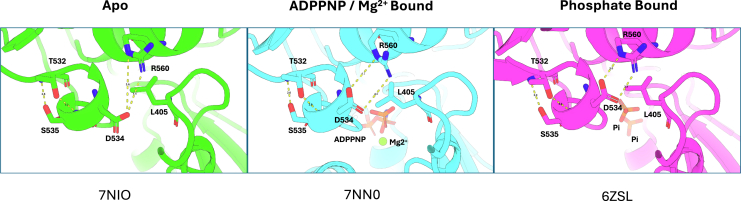
**Interaction of D534 with R560 is dependent on phosphate binding in the NTPase binding site**. Motif V and adjacent interacting residues are shown from nsp13 in the apo form (PDB:7NIO), nsp13 bound to ADPPNP and Mg^2+^ modeling the pre-ATP hydrolysis step (PDB: 7NN0), and phosphates modeling the post-ATP hydrolysis step (PDB: 6ZSL).

Kinetic characterization of the SARS-CoV-2 nsp13 helicase thus far has provided a baseline of helicase behavior and has aided in the development of small molecule inhibitors against helicase activity (33, 34). Studies to date on nsp13 helicase kinetics have focused primarily on nucleic acid interactions with the binding domain, with little attention to the linkage between ATP hydrolysis and helicase function (33, 35, 36). Previous work on Motif V in flaviviruses link energy transduction from the ATPase domain to the helicase domain, but so far, this has only been explored in theoretical computational models for SARS-CoV-2 (30). In this work, we leverage these simulation data to characterize the SARS-CoV-2 nsp13 Motif V and demonstrate residue specific interactions through biochemical kinetic assays. We developed steady state kinetic assays independently examining ATPase and helicase activities and examined the effect of nsp13 mutants within and proximal to Motif V on helicase and ATPase activity as well as substrate binding to develop an enzyme kinetic model that allows us to approximate the kinetic steps influenced by Motif V. Nsp13 Motif V mutations T532A and S535A disrupted a critical hydrogen bond that stabilizes the secondary structure of Motif V and significantly increased both ATPase activity and helicase activity, suggesting a negative regulatory role of these residues in nucleic acid unwinding. We also explored the D534 residue that links Motif V to the helicase domain and found both direct mutation to alanine and indirect disruption through a neighboring L405D mutation nearly abolished helicase activity while retaining ATPase activity. These data validate previous computational data indicating the importance of Motif V in ATPase-to-helicase crosstalk in nsp13 helicase function and demonstrate that Motif V serves as a crucial link between the SARS-CoV-2 nsp13 helicase ATPase and helicase active sites.

## Results

Previous work in our group found that Motif V plays a role in communication between the ATPase domain and the RNA unwinding helicase domain in the flavivirus NS3 helicase (32). The coronavirus nsp13 Motif V spans amino acid residues 530 to 544 and is highly conserved across other human pathogenic coronaviruses (Fig. 1*C*). Analysis of the crystal structure of nsp13 revealed a helical secondary structure including a completely conserved TVDS sequence motif between amino acids 532 and 535, stabilized by a hydrogen bond between T532 and S535, similarly reported in NS3 (Fig. 2) (32). Motif V spans between the RNA binding site and stretches into the ATP binding domain, making a hydrogen bond with bound ATP between ribose O3 and E540, with the residues involved in the helical T532-S535 structure contributing to the allosteric communication between the two domains. The highly conserved nature of Motif V across the betacoronavirus family indicates the motif’s importance in enzymatic function. Given our previous experience with Motif V in RNA virus helicases, we sought to understand more about the contributions of these residues to enzymatic activity.

Recombinant proteins containing point mutations at desired Motif V residues were mutated and purified to test activity. Single-point mutants at T532 and S535 were chosen based on the structural homology of Motif V to the flavivirus NS3 helicase protein, which had previously been shown to be involved in allosteric communication between the NTPase and helicase domains of NS3 (32). All purified mutations were not within the active sites of either domain; however, we wanted to make sure mutations did not affect the structure of the proteins and the substrate binding. Thermal shift assays using hydrophobic dye, Sypro Orange, indicated structural integrity did not change significantly between mutations (Fig. S1*D*). Dissociation constants (K_d_) of dsDNA were determined using fluorescence anisotropy measurements with a fluorescently labeled dsDNA and nsp13 mutants to check if mutations affected dsDNA binding. DNA substrates contain a 5′ overhang of single-stranded DNA that is required for helicase initiation and is incorporated in kinetic assays (25). While the binding mode of DNA substrate to purified helicase is not clear in regard to preference toward the single-stranded or the double-stranded region, the DNA used in binding assays contained the single-stranded overhang for consistent understanding of the helicase interaction with the substrates on which kinetic analyses were measured. Beta-coronaviruses are single-stranded RNA viruses, and their replication generates double-stranded RNA intermediates. However, nsp13 has been found to have comparable activities with double-stranded DNA and RNA/DNA substrates, making use of more stable dsDNA in binding and unwinding assays attractive (10).

**Figure 3:**
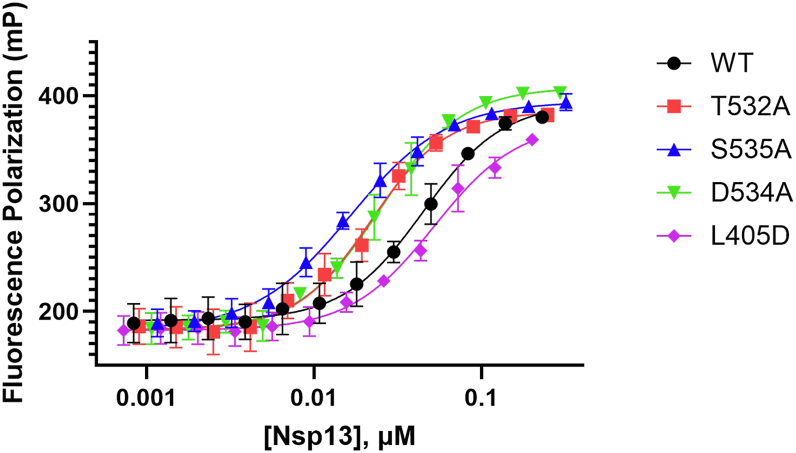
**Fluorescence polarization analysis of nsp13 mutant DNA-binding affinity**. Fluorescence polarization was used to determine binding affinity for each mutant. Nsp13 WT and designated mutants were titrated into reactions with 10 nM 5′-labeled AlexaFluor 488-dsDNA. Fluorescence polarization values represent the fraction of labeled dsDNA bound to nsp13. K_d_ values were derived from a four-parameter logistic curve fit using Graphpad Prism 10 and are reported in Table 1. n = 3.

The nsp13 T532A and S535A mutants bound dsDNA approximately two-fold tighter than WT nsp13 (Fig. 3). In addition to these mutations, we wished to understand if the interposed residue, D534, played a role in the stability of Motif V, at the suggestion of the molecular dynamics simulations in previously published works (30). Motif V was thought to be important in the allosteric communication between the ATP-binding site and the RNA-binding cleft, with D534 noted for its power stroke-like movement across sampled conformational clusters. The L405D residue was proposed to exhibit a repulsive effect on D534 and further probe the importance of Motif V on enzyme activity (30). Similar to T532 A and S535 A, D534 A bound dsDNA approximately two-fold more tightly than WT, while the K_d_ of L405D did not change significantly (Table 1). The DNA-binding data confirm that mutations did not affect the ability of nsp13 mutants to bind dsDNA substrate, and thermal shift data corroborate that further analysis of steady-state kinetics is not a result of structural disruptions caused by the introduced mutations (Fig. 3).

**Table 1:** Binding affinity of WT and mutant nsp13 for dsDNA substrates

Nsp13 variant	WT	T5322 A	S535 A	D534 A	L405D
K_d_ (nM ± SE)	45 ± 4	21 ± 1∗∗	17 ± 3∗∗	26 ± 5∗	52 ± 1^ns^

Binding assays carried out in 50 mM HEPES, pH 7.50 buffer with 2 mM DTT, 0.01% NP-40, 10 nM AlexaFluor 488-labeled dsDNA, and varied nsp13 concentrations. n = 3, ∗∗*p* ≤ 0.01, ∗*p* ≤ 0.05, ns = no significance.

**Figure 4:**
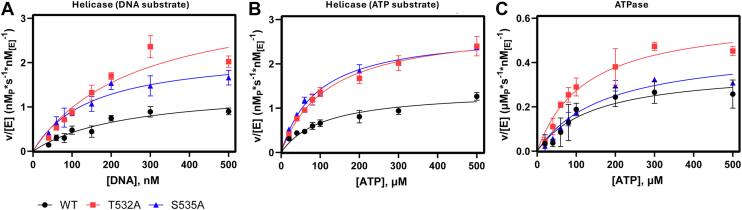
**Michaelis-Menten kinetics enzymatic characterization of nsp13 T532A and S535A mutants**. *A*, reactions were carried out in a 20 mM HEPES, pH 7.50 reaction buffer with 20 mM NaCl, 5 mM MgCl_2_, 1 mM DTT, 0.1% bovine serum albumin, and 1 nM of WT (*black circle*), T532 A (*red square*) or S535 A (*blue up triangle*) nsp13. *A*, Helicase kinetic characterization using DNA as the variable substrate. Reactions were incubated with 40–500 nM dsDNA with a AlexaFluor 488/IowaBlack fluorophore-quencher pair, and initiated with 500 μM ATP. *B*, Helicase activity characterization using ATP as the variable substrate. Reactions were incubated with100 nM labeled dsDNA and initiated with 20–500 μM ATP. Initial rates were determined as a function of increase fluorescence, RFU values were converted to ssDNA concentration, and product concentration per second rate units were normalized to enzyme concentration. Plotted values are nM ssDNA product per second per nM enzyme. *C*, ATP activity characterization. Reactions were incubated with100 nM unlabeled dsDNA and initiated with 20 to 500 μM ATP. Initial rates were determined through the colorimetric BioMOL Green assay to detect free phosphate concentration. Absorbance values were converted to [P_i_], and rate data was plotted similarly to helicase data, with rate values in μM phosphate product per second per nM enzyme. K_cat_, K_m_, and K_cat_/K_m_ values are reported in Table 2, Table 3, Table 4, respectively. n ≤ 3.

The regulatory nature of nsp13 Motif V is hypothesized to be driven by strong hydrogen bond between T532 and S535, therefore analyzing the mutational effects on steady state kinetics of both the ATPase and the helicase domains provide an understanding of the kinetic propagation driving ATPase/helicase crosstalk. Helicase assays were developed using a previously established method using a fluorophore/quencher-paired double stranded oligonucleotides. Of note, MgCl_2_ concentration was unchanged from the original protocol at 5 mM and was in excess of ATP. Excess Mg^2+^ can have inhibitory effects, however, the activity of the enzyme at the given concentration was sufficient for our work. Mutations in Motif V were in a different enzymatic domain to established ion binding residues and second sphere aquo water interactions, therefore [Mg^2+^] was not futher optimized for our WT or mutant enzymes. Equilibrated helicase reactions were initiated by the addition of ATP, and initial rate was determined as a function of product formation *via* unwound fluorophores separating from the quencher strand. From initial rates, k_cat_ provides information on the effects of the mutations on the rate determining step, ATP hydrolysis, K_M_ indicates the substrate concentration at half ν_max_, and combining these constants to get k_cat_/K_M_ describes the catalytic efficiency of the enzyme and how point mutations affect the ability for substrate to bind and undergo enzymatic catalysis (37). Michaelis–Menten kinetics of T532A (k_cat_ = 3.7 ± 0.6 s^-1^, k_cat_/K_M_ = 0.014 ± 0.004 s^-1^∗nM_[E]_^-1^) and S535A (k_cat_ = 2.4 ± 0.3 s^-1^, k_cat_/K_M_ = 0.016 ± 0.006 s^-1^∗nM_[E]_^-1^) as DNA substrate concentration varies revealed an increase in k_cat_ and k_cat_/K_M_ compared to WT (k_cat_ = 1.5 ± 0.2 s^-1^, k_cat_/K_M_ = 0.006 ± 0.002 s^-1^∗nM_[E]_^-1^), however only changes in T532A were statistically significant (Fig. 4*A*). When ATP substrate was varied, the kinetic effect on k_cat_ and k_cat_/K_M_ also significantly increased in T532A (k_cat_ 2.9 ± 0.3 s^-1^, k_cat_/K_M_ = 0.024 ± 0.004 s^-1^∗nM_[E]_^-1^) and S535A (k_cat_ 2.8 ± 0.2 s^-1^, k_cat_/K_M_ = 0.030 ± 0.002 s^-1^∗nM_[E]_^-1^) relative to WT (k_cat_ 1.4 ± 0.2 s^-1^, k_cat_/K_M_ = 0.013 ± 0.004 s^-1^∗nM_[E]_^-1^) (Fig. 4*B* and Table 3). Taken together, the effect of mutating either hydrogen-bond contributor within Motif V increases both the unwinding rate of the helicase domain, and the enzymatic efficiency, as the change in rate is not significantly reflected in the K_M_.

**Table 2:** Michaelis Menten data for helicase with DNA substrate^a^

Nsp13	K_cat_ (s^-1^)	K_M_ (nM)	K_cat_/K_M_ (s^-1^∗nM^-1^)
WT	1.5 ± 0.2	285 ± 86	0.006 ± 0.002
T532 A	3.7 ± 0.6∗	282 ± 77^ns^	0.014 ± 0.004∗
S535 A	2.4 ± 0.3^ns^	175 ± 63^ns^	0.016 ± 0.006^ns^

n = 3, ∗∗p ≤ 0.01, ∗p ≤ 0.05, ns = no significance.

a Helicase assays carried out in 20 mM HEPES, pH 7.50, 20 mM NaCl, 5 mM MgCl_2_, 1 mM DTT, 0.1% bovine serum albumin, and 1 nM enzyme. Table 2 reactions prepared with fluorophore quencher paired dsDNA varied concentration and initiated with 500 μM ATP.

**Table 3:** Michaelis Menten data for helicase with ATP substrate^a^

Nsp13	K_cat_ (s^-1^)	K_M_ (μM)	K_cat_/K_M_ (s^-1^∗μM^-1^)
WT	1.4 ± 0.2	122 ± 34	0.013 ± 0.004
T532 A	2.9 ± 0.3∗	121 ± 16^ns^	0.024 ± 0.004∗
S535 A	2.8 ± 0.2∗∗	94 ± 8^ns^	0.030 ± 0.002∗∗

n = 3, ∗∗p ≤ 0.01, ∗p ≤ 0.05, ns = no significance.

a Helicase assays carried out in 20 mM HEPES, pH 7.50, 20 mM NaCl, 5 mM MgCl_2_, 1 mM DTT, 0.1% bovine serum albumin, and 1 nM enzyme. Table 3 reactions prepared with100 nM labeled dsDNA and initiated with varied concentrations of ATP.

Considering the kinetics as a whole, the reaction coordinate of the helicase involves two active sites, the ATPase domain where ATP is hydrolyzed, the helicase domain where DNA is unwound, and the cross-talk between the two. The role of the hydrogen bond between T532 and S535 is consistent with that observed previously in flaviviral NS3 helicase, in that they appear to regulate cross talk between the ATPase and helicase domain. The increased binding of DNA with both mutants is reflected in the turnover rate and therefore the enzymatic efficiency in the helicase when DNA concentration is varied. Because ATP hydrolysis is in a disconnected active site and helicase activity was affected in T532 A and S535 A mutants when ATP was varied, the hydrogen bond in Motif V is a regulator in ATPase-to-helicase crosstalk.

Varying ATP led to kinetic changes in the helicase domain in T532A and S535A movements; the ATPase active site was subjected to kinetic analyses using the malachite green-based BioMOL Green assay. Initial rate data were acquired as a function of product formation *via* phosphate release and were initiated by adding varied concentrations of ATP to an equilibrated reaction. Once the reaction was initiated, aliquots of the reaction were quenched in malachite green solution, which halted enzyme activity and started developing phosphate detection, and the colorimetric change was quantified using a plate reader (Fig. 4*C* and Table 4). The k_cat_ values of T532A and S535A increased (0.61 ± 0.03 s^-1^ and 0.47 ± 0.02 s^-1^, respectively) compared to WT (0.37 ± 0.02 s^-1^). The k_cat_/K_M_ of S535A was unchanged compared to WT, but T532A was increased. However, there was no statistical significance (*p* ≤ 0.05) between the wild type and T532A and S535A mutant k_cat_/K_M_, indicating that the mutants are affecting the turnover rate but not enzymatic efficiency (Table 4). Motif V residues extend into the ATPase domain and interact with bound ATP, and removing the T532-S535 hydrogen bond likely provides more flexibility to elongate Motif V into the ATPase domain. The mutations only significantly increase k_cat_ values, indicating the mutations affect the rate-limiting hydrolysis step, and not the prior binding steps.

**Table 4:** Michaelis Menten data for ATPase^a^

Nsp13	K_cat_ (s^-1^)	K_M_ (μM)	K_cat_/K_M_ (s^-1^∗μM^-1^)
WT	0.37 ± 0.02	156 ± 30	0.0027 ± 0.0008
T532 A	0.61 ± 0.03∗∗	126 ± 20^ns^	0.0050 ± 0.0006^ns^
S535 A	0.47 ± 0.02∗	176 ± 17^ns^	0.0027 ± 0.0002^ns^

n = 3, ∗∗*p* ≤ 0.01, ∗*p* ≤ 0.05, ns = no significance.

a ATPase assays carried out in 20 mM HEPES, pH 750, 20 mM NaCl, 5 mM MgCl_2_, 1 mM DTT, 0.1% bovine serum albumin, 1 nM enzyme, 100 μM dsDNA, and initiated with varied concentrations of ATP.

**Figure 5:**
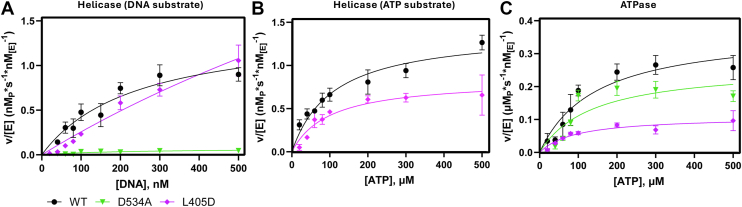
**Michaelis-Menten Kinetics Enzymatic Characterization of nsp13 L405D and D534 A Mutants**. Reactions were carried out in a 20 mM HEPES, pH 7.50 reaction buffer with 20 mM NaCl, 5 mM MgCl_2_, 1 mM DTT, 0.1% bovine serum albumin, and 1 nM of WT (*black circle*), D534 A (*green down triangle*) or L405D (magenta diamond) nsp13. *A*, Helicase kinetic characterization using DNA as the variable substrate. Reactions were incubated with 40–500 nM dsDNA with a AlexaFluor 488/IowaBlack fluorophore-quencher pair and initiated with 500 μM ATP. *B*, Helicase activity characterization using ATP as the variable substrate. Reactions were incubated with100 nM labeled dsDNA and initiated with 20–500 μM ATP. Given the lack of helicase activity for D534 A in panel A, helicase activity for D534 A was not determined using ATP as the variable substrate. Initial rates were determined as a function of increase fluorescence, RFU values were converted to ssDNA concentration, and product concentration per second rate units were normalized to enzyme concentration. Plotted values are nM ssDNA product per second per nM enzyme. *C*, ATP activity characterization. Reactions were incubated with100 nM unlabeled dsDNA and initiated with 20 to 500 μM ATP. Initial rates were determined through the colorimetric BioMOL Green assay to detect free phosphate concentration. Absorbance values were converted to [P_i_], and rate data was plotted similarly to helicase data, with rate values in μM phosphate product per second per nM enzyme.K_cat_, K_m_, and K_cat_/K_m_ values are reported in Table 2, Table 3, Table 4, respectively. n ≤ 3.

As mentioned prior, molecular dynamic simulations performed on nsp13 found that Motif V residue D534 seems to exhibit a conformationally driven power stroke movement, stabilized by R560 (also in subdomain 2A) (30). Given the molecular dynamic simulation data available, its high conservation, and its proximity to T532 and S535, we hypothesized that D534 play a role in stabilizing the Motif V secondary structure and participating in the allosteric crosstalk between the ATPase and helicase. Michaelis-Menten kinetic analysis of the helicase domain revealed the D534 A mutation nearly abolished all helicase activity when DNA concentration was varied (Fig. 5*A*). Due to the low activity at high DNA concentrations, Michaelis-Menten kinetics were not collected with a variance of ATP, as the measured range of ATP would be less than that of the constant concentration of 1 mM used when measuring varied DNA; therefore, we inferred that any Michaelis-Menten constants derived would be significantly reduced (Fig. 5*B*). To perturb the interaction of D534 and R560, an L405D mutation was made (situated by the D534 and R560 residues) that would repel the D534 and compete with the R560 interaction. The L405D mutant was still active but increased both the k_cat_ and the K_M_ values in the helicase domain when DNA was varied (Table 5). The fit K_M_ value exceeded the range of measured DNA concentration, therefore k_cat_/K_M_ was derived from a modified fit of the Michaelis-Menten equation for sub-saturating conditions ([S]<<K_M_) (37). Enzymatic efficiency of DNA unwinding activity in the helicase for the L405D mutant was not significant compared to the WT enzyme (k_cat_/K_M_ = 0.003 ± 0.002 and 0.006 ± 0.001 s^-1^∗nM^-1^, respectively). When ATP was varied, helicase activity was observed, but Michaelis-Menten constants were not significantly altered (Table 6).

**Table 5:** Michaelis Menten data for helicase with DNA substrate^a^

Nsp13	K_cat_ (s^-1^)	K_M_ (nM)	K_cat_/K_M_ (s^-1^∗nM^-1^)
WT	1.5 ± 0.2	285 ± 86	0.006 ± 0.002
D534 A	n.d.	n.d.	n.d.
L405D	10 ± 6^ns^	>500	0.003 ± 0.002^ns^

n = 3, ∗∗p ≤ 0.01, ∗p ≤ 0.05, ns = no significance.

a Helicase assays carried out in 20 mM HEPES, pH 7.50, 20 mM NaCl, 5 mM MgCl_2_, 1 mM DTT, 0.1% bovine serum albumin, and 1 nM enzyme. Table 5 reactions prepared with fluorophore quencher paired dsDNA varied concentration and initiated with 500 μM ATP.

**Table 6:** Michaelis Menten data for helicase with ATP substrate^a^

Nsp13	K_cat_ (s^-1^)	K_M_ (μM)	K_cat_/K_M_ (s^-1^∗μM^-1^)
WT	1.4 ± 0.2	122 ± 34	0.013 ± 0.004
L405D	0.9 ± 0.2^ns^	118 ± 50^ns^	0.010 ± 0.005^ns^

n = 3, ∗∗p ≤ 0.01, ∗p ≤ 0.05, ns = no significance.

a Helicase assays carried out in 20 mM HEPES, pH 7.50, 20 mM NaCl, 5 mM MgCl_2_, 1 mM DTT, 0.1% bovine serum albumin, and 1 nM enzyme. Table 5 reactions prepared with fluorophore quencher paired dsDNA varied concentration and initiated with 500 μM ATP. Table 6 reactions prepared with100 nM labeled dsDNA and initiated with varied concentrations of ATP.

Both the L405D and D534 mutants were still able to hydrolyze ATP substrate. The k_cat_ and k_cat_/K_M_ for D534 A (0.26 ± 0.03 s^-1^ and 0.0019 ± 0.0003 s^-1^ nM^-1^, respectively) were not significantly lower than WT (0.37 ± 0.02 s^-1^ and 0.0027 ± 0.0008 s^-1^∗nM^-1^, respectively) whereas k_cat_ of L405D (0.14 ± 0.05) was reduced (Fig. 5*C*, Table 7). This further highlights the dependence on Motif V for chemical energy produced during ATP hydrolysis being directed to the helicase domain, and the critical role D534 plays in facilitating that movement. The D534A mutant did not affect ATPase activity, and increased DNA binding affinity to the helicase domain, but DNA procession through the helicase was unable to occur. By adding a competitor for the D534:R560 interaction with the L405D mutant, helicase and ATPase activity was still observed, although that driving force from one active site to the other was attenuated.

**Table 7:** Michaelis Menten data for ATPase^a^

Nsp13	K_cat_ (s^-1^)	K_M_ (μM)	K_cat_/K_M_ (s^-1^∗μM^-1^)
WT	0.37 ± 0.02	156 ± 30	0.0027 ± 0.0008
D534 A	0.26 ± 0.03^ns^	135 ± 12^ns^	0.0019 ± 0.0003^ns^
S535 A	0.14 ± 0.05∗	162 ± 97^ns^	0.0013 ± 0.0009^ns^

a ATPase assays carried out in 20 mM HEPES, pH 750, 20 mM NaCl, 5 mM MgCl_2_, 1 mM DTT, 0.1% bovine serum albumin, 1 nM enzyme, 100 μM dsDNA, and initiated with varied concentrations of ATP. n = 3, ∗∗*p* ≤ 0.01, ∗*p* ≤ 0.05, ns = no significance.

## Discussion

This study provides an extensive kinetic analysis of the α-helical structure of nsp13 Motif V and its role in regulating energy transduction from ATP hydrolysis in the nsp13 NTPase active site to the nsp13 helicase active site, where bound double-stranded nucleic acids are unwound. We have previously studied a similar regulatory mechanism of Motif V in the flavivirus helicase, NS3, and found that SARS-CoV-2 helicase nsp13 contains a comparable structural scaffold made up of a highly conserved TVDS motif also found across other human pathogenic coronaviruses. We found that mutating the T532 and S535 residues to alanine groups disrupted a hydrogen bond formed between the two, resulting in an increase in nsp13 helicase activity, ATPase activity, and ATP-to-helicase crosstalk. Conversely, mutation of the Motif V D534 residue abolished helicase activity but did not affect ATPase activity. This effect was further validated with an L405D mutant that repulsed D534 and competed for interaction with D560, which rescued some helicase activity and ATP-to-helicase crosstalk with attenuated efficiency and decreased ATP hydrolysis turnover in the ATPase site.

**Figure 6:**
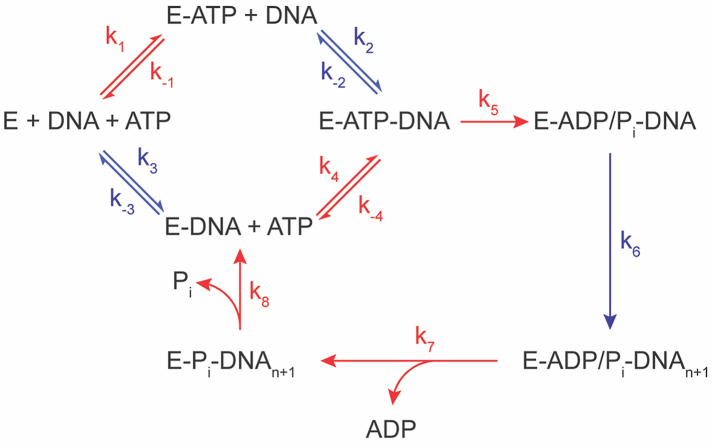
**Model for the nsp13 enzymatic mechanism**. Proposed mechanism of the kinetically-coupled active sites in nsp13 through a single turnover of ATP and the procession of one nucleotide step. Kinetic steps occurring in the ATPase active site are in red, and steps occurring in the helicase domain are in *blue*.

The proposed kinetic reaction diagram of the helicase requires examining enzyme activity using two active sites, with kinetic steps occurring in the ATPase domain in red, and steps occurring in the helicase domain in blue (Fig. 6). From apoenzyme, either ATP or DNA can reversibly bind the enzyme (k_1_/k_-1_ or k_3_/k_-3_, respectively), followed by the second substrate (k/k_-2_ for DNA to E-ATP or k_4_/k_-4_ for ATP to E-DNA). Once all substrates are bound, the first irreversible step is the hydrolysis of ATP, wherein k_5_ in our model may include both the rate-limiting conformational change and the hydrolysis step. The hydrolysis of ATP drives a conformational change believed to alter RNA binding in specific enzymatic domains to drive the DNA strand through the helicase domain (k_6_). The subsequent release of ADP, followed by P_i_ (k_7_ and k_8_, respectively), is believed to be ordered by sterics and returns the enzyme to a DNA-bound enzyme ready to accept another ATP molecule (38).

In this study, DNA is bound to the enzyme prior to reaction initiation with ATP and therefore proceeds primarily through the k_3_/k_4_ pathway. Binding assays conducted with DNA in the presence and absence of ATP demonstrated that DNA binds more tightly prior to ATP binding, consistent with previously reported DNA activation of the ATPase site (39). Furthermore, the nsp13 ATPase domain had no activity when DNA was not bound; therefore, our data are interpreted with focus on the lower pathway (Fig. S4).

The increase in catalytic turnover and enzymatic efficiency in the helicase domain of T532A and S535A mutant enzymes can likely be partially attributed to the tighter binding of DNA to the enzyme (K_d_) as is reflected in the increase in k_cat_/K_M_. In the ATPase assay, the k_cat_/K_M_ was not significantly affected, but the k_cat_ was increased, suggesting the broken hydrogen bond increased k_5_ by allowing Motif-V residues more flexibility to stabilize the hydrolyzed products in the ATPase active site. The effect of ATP hydrolysis on the helicase activity is ascertained from the helicase kinetic assay, where ATP is varied. The increased k_cat_ observed with T532 A and S535 A (Tables 2–7) highlights the proficiency with which the T532 A and S535 A direct hydrolysis energy to the helicase and shows the increased k_5_ from the ATPase domain is also represented in helicase kinetic constant, k_6_ as expected of a subsequent step to the rate-limiting step. The k_cat_/K_M_ is expressed as an apparent second order rate constant to the helicase activity as a result of varied ATP concentrations, which occurs in separate active sites. However, it still respresents the pre-irreversible substrate interactions and provides kinetic data on conformationally driven active site communication rather than traditional turnover chemistry. The pre-equilibrium step that likely leads to increased k_cat_/K_M_ carries over from the increased enzymatic efficiency from the DNA varied helicase assay.

Contrary to our observations with the T532A/S535A mutants, the D534 A mutation abolished helicase activity and L405D attenuated activity. Interestingly, the decreases in observed Michaelis-Menten constants in the D534 A mutant did not occur in the ATPase assay. The required DNA activation of the ATPase domain and the decreased dsDNA K_d_ of D534 A indicated that the mutation did not affect ATP hydrolysis in the ATPase domain. Therefore, the abolition of helicase activity represents a decrease in k_6_ as the energy barrier to drive DNA through the binding cleft is too high, implicating the importance of D534 in the conformational stabilization of Motif V in ATPase-to-helicase crosstalk. The L405D mutant further demonstrated this by indirectly disrupting the previously proposed D534-R560 salt bridge that drives a power stroke motion of the DNA binding cleft (30). The K_d_ of dsDNA to L405D was similar to WT, however the increase in K_M_ and unchanged catalytic efficiency show that bound dsDNA struggles to unwind, and the reversible dsDNA binding steps prior to k_5_ that comprise K_M_ become more apparent. The hydrolysis of ATP is also affected by L405D, as indicated by the decrease in k_cat_, potentially due to repulsion of D534 limiting the flexibility of Motif V into the ATPase domain.

The coupling of the ATPase domain to helicase unwinding has been widely noted in Motif III of bacterial helicases, but no consistent model exists for how the active sites depend on each other, nor has Motif V (which like Motif III also extends into both active sites) been as widely studied. The ATPase activity is conformationally restricted without ssRNA present. Once bound, the active site is opened, but the degree of translocation per hydrolysis differs across helicases (40). In bacterial helicases, the *Escherichia coli* helicases RecQ and UvrD observed two- to five-nucleotide advancement to one ATP hydrolysis event, respectively (41, 42). ATPase to helicase coupling was disrupted in RecQ by mutating aromatic W154, F158, and R159 to leucine in Motif III, which resulted in a reduction in unwinding, while ssRNA binding and ATP hydrolysis were unaffected (43). In *Bacillus stearothermophilus*, motif III in PcrA helicase was found to couple ATP hydrolysis to DNA unwinding through kinetic findings that Q260A and Q254A mutations decoupled helicase and ATPase activities despite DNA binding being affected (44). In viral helicases, the effects of Motif V on helicase activity have been better characterized. Mutation of Motif V T809 and G815 in HSV UL5 diminished ATPase and helicase activity, structural analysis of HCV NS3 showed conformational changes in Motif V residues 414 to 419 that influence ATP binding, and biochemical analysis, previously conducted by our group on flavivirus NS3 helicase, demonstrated a regulatory role of Motif V residues T407 and S411 (32, 45, 46, 47).

In conclusion, we present experimental data that helps validate both structural and computational hypotheses about how Motif V serves as a conformational link between the nsp13 NTPase and helicase active sites and expands on the existing findings on the role of Motif V in coupling ATPase hydrolysis to nucleotide unwinding through extensive steady-state kinetic analysis on key Motif V-associated residues. These data will be useful in further exploration of the molecular mechanisms that nsp13 and other related RNA helicases use to unwind nucleic acids and may provide valuable insights for future antiviral therapeutic and vaccine development.

## Experimental procedures

### Nsp13 mutant cloning

All point mutations were made on a plasmid containing the gene encoding N-terminally 6xHis-ZBasic-tagged SARS-CoV-2 helicase, nsp13 (Addgene plasmid # 159614). Motif V mutations were made *via* Q5 site-directed mutagenesis with forward primers flanking the desired mutation site and reverse primers beginning at the 5′ end of the forward primer to generate a linear product, found in Table S1. PCR products were amplified using Q5 High Fidelity Polymerase (New England Biolabs, Cat No. M0491), following manufacturer specifications, and modifying the thermocycling protocol to a touchdown PCR protocol from 68 °C to 58 °C in increments of 1 °C per cycle during the annealing stage of the first 10 cycles, followed by a 60 °C annealing stage for the remaining 25 cycles. Linear PCR products were gel-purified and extracted using the Zymoclean Gel DNA recovery kit (Zymo Research, Cat. No. D4001), circularized with a KLD enzyme mix (New England Biolabs), and transformed into *E*. *coli* Stbl3 competent cells (Invitrogen, Cat. No. C737303). Clones were selected for kanamycin resistance on LB-Agar plates and grown in 5 ml LB media supplemented with 50 μg/ml kanamycin. Plasmids were extracted with QIAprep Spin Miniprep Kit according to manufacturer’s protocol (Qiagen, Cat. No. 27104), and point mutations were validated with whole plasmid sequencing (Azenta).

### Nsp13 expression and purification

Nsp13 proteins were expressed and purified as previously described (19). Briefly, the pNIC-ZB 6xHis-ZB-SARS-CoV-2 nsp13 plasmid or mutated variants were transformed into *E*. *coli* BL21 Rosetta2 competent cells and selected for kanamycin resistance on LB-agar plates. Single colonies were picked and grown in 50 ml Terrific Broth supplemented with 50 μg/ml kanamycin overnight at 37 °C and 180 rpm, then 1 ml inoculum were added 500 ml Terrific Broth media with 50 μg/ml kanamycin at 37 °C and 180 rpm to an OD_600_ of 2. Protein expression was induced with 300 μM IPTG and shaken overnight at 18 °C at 180 rpm. Bacteria were collected and resuspended in lysis buffer (50 mM HEPES, pH 7.50, 500 mM NaCl, 5% glycerol, 10 mM imidazole, 500 μM TCEP) with protease inhibitor cocktail (ThermoFisher Pierce, Cat No. A32955) prior to storage at −80 °C. For protein purification, bacteria were thawed and lysed on wet ice *via* probe sonication at 20% amplitude for five 3-min rounds of 10 s on/5 s off. Lysates were clarified at 30,000 x G for 45 min at 4 ^o^C, supernatants were sterile filtered through a 0.45 μm membrane and loaded at 1 ml/min onto a HisTrap HP 5 ml nickel column (Cytiva) pre-equilibrated with low imidazole buffer (50 mM HEPES, pH 7.50, 500 mM NaCl, 5% glycerol, 10 mM imidazole, 500 μM TCEP) on an AKTA Start FPLC system. The column was washed with five column volumes (CVs) at 2 ml/min of wash buffer (50 mM HEPES, pH 7.5, 500 mM NaCl, 5% glycerol, 45 mM imidazole, 500 μM TCEP), followed by 2 CVs at 2 ml/min of high salt buffer (50 mM HEPES, pH 7.5, 1 M NaCl, 5% glycerol, 500 μM TCEP). The column was washed with 5 CVs at 2 ml/min of wash buffer, then eluted with 5 CVs at 2 ml/min of high imidazole buffer (50 mM HEPES, pH 7.5, 500 mM NaCl, 5% glycerol, 300 mM imidazole, 500 μM TCEP, Fig. S1A). Eluted protein was concentrated to <1 ml in a protein PES concentrator tube with 30K MWCO (ThermoFisher Pierce Cat No. 88531) and loaded onto a HiLoad 16/600 Superose 6 pg size exclusion column (Cytiva) pre-equilibrated with running buffer (50 mM HEPES, pH 7.5, 500 mM NaCl, 5% glycerol, 500 μM TCEP, Fig. S1B). Samples were resolved at 1 ml/min with running buffer, peaks containing full-length nsp13 identified by SDS-PAGE and combined, concentrated to <1 ml in a protein concentrator tube with 30K MWCO and concentrated samples stored at −80 °C in single-use aliquots (Fig. S1C). Purified protein samples were characterized for purity with 12% SDS-PAGE gel, concentration with Qubit Protein BR Assay on Qubit3 Fluorometer, and assessed for structural stability (Fig. S1D) with a thermal shift assay using Protein Thermal Shift dye kit (ThermoFisher)

### Helicase assay

Nsp13 helicase function was assessed using a fluorescence dequenching method as previously described (48). This paper found that dsDNA unwinding was stronger than dsRNA unwinding for nsp13 for enzymatic analyses, so we opted to use dsDNA unwinding to assess nsp13 helicase function. Briefly, fluorophore/quencher-paired dsDNA was prepared fresh to 10 μM with single stranded 5′-AlexaFluor488 oligonucleotide (BG1713) and 3′-IowaBlack oligonucleotide (BG1714, Table S1) in ultrapure water and incubated at 95 °C for 3 min (48). To initiate a helicase reaction, a 1.25x 400 μl DNA reaction solution was prepared with 2x buffer (40 mM HEPES (pH 7.50), 40 mM NaCl, 10 mM MgCl_2_, 2 mM DTT, 0.2% bovine serum albumin), 1.25 nM nsp13, and 250 nM BG1713/1714 or a DNA concentration indicated within each figure. A 5x 100 μl ATP initiation solution was prepared with 2x buffer, 2.5 μM of competitor ssDNA (BG1715), and 2.5 mM ATP or an ATP concentration indicated within each figure. The 1.25x DNA reaction solution was added into a quartz cuvette, mounted into a water-jacketed Edinburgh FS5 spectrofluorometer and equilibrated to 37 °C for 2 minutes. Fluorescence monitoring was conducted with Fluoracle software at 495 nm excitation, 516 nm emission, measuring every 0.5 s for 60 s. After 20 s, DNA unwinding was initiated with the addition of 100 μl of 5x ATP solution to the cuvette, and initial rate data were collected over the remaining 40 s. Standard curves were generated to correlate relative fluorescence units (RFU) to DNA concentration, and initial rates were determined *via* linear fit on data collected using the first 5 s after ATP initiation (Fig. S2). Michaelis-Menten curves were fit to initial rate data using Prism GraphPad 10 to derive k_cat_, K_M_, and k_cat_/K_M_.

### Nsp13:dsDNA K_d_ determination

Fluorescence polarization was used to calculate dissociation constants between nsp13 and nucleic acids as previously reported (32). Briefly, purified nsp13 was diluted 1.5:1 in size exclusion running buffer for a total of 12 concentrations, and 10 μl of each were added a black 384-well microplate, followed by 40 μl of a 1.25x binding buffer (50 mM HEPES pH 7.50, 2.5 mM DTT, 0.0125% NP-40, 12.5 nM dsDNA (BG1713/1715), to a final concentration of 50 mM HEPES, pH 7.50, 2 mM DTT, 0.01% NP-40, and 10 nM dsDNA (BG1713/1715), and 100 mM NaCl, 1% glycerol, 100 μM TCEP contribution from Nsp13 buffer. Concentration ranges of Nsp13 were based on initial concentrations of purified protein, and the stoichiometric range of Nsp13 to dsDNA substrate varied from 0.1:1 to 30:1 for binding curve collection. Fluorescence polarization values were measured on a Victor X5 multimode plate reader (PerkinElmer), and data were fit to a variable slope model, four-parameter logistic function using Graphpad Prism 10 to determine K_d_.

### ATPase assay

We used a colorimetric malachite green-based assay to detect free phosphate generated by nsp13-mediated hydrolysis of ATP to ADP (32). Reactions and buffers were prepared similarly to the helicase reaction. Double-stranded DNA was prepared with 10 μM unlabeled oligonucleotides (BG2261/2262) in 2x reaction buffer. To run an ATPase assay, a 1.25x DNA reaction solution was prepared with 2x reaction buffer, 250 nM dsDNA, and 1.25 nM nsp13 and warmed to 37 °C. Individual 5x ATP initiation solutions were prepared with 2x buffer at 100 μM, 200 μM, 300 μM, 400 μM, 500 μM, 1 mM, 1.5 mM, or 2.5 mM ATP. To a 96-well flat-bottom clear microplate placed on a 37 °C heat block, 320 μl of 1.25x DNA reaction solution was added to all column 1 wells, and 100 μl BIOMOL Green (Enzo Life Sciences, Cat No. BML-AK111) was added to wells in columns 2 through 6. Using a programmable electronic 8-channel pipette, reactions were initiated by adding 80 μl of 5x ATP initiation solutions into column 1 wells. From the reacting solution, 300 μl was drawn into the automatic pipettor, where the reaction continued in the pipette tip, and timepoint data was gathered by quenching 50 μl of the reaction mix into well two through six containing 100 μl BIOMOL Green at 3 s increments for 15 s to quench the reaction. On the same plate, a standard curve of inorganic phosphate was generated following the BIOMOL Green manufacturer’s protocol with provided phosphate standards. Plates were incubated at 22 °C for 30 min, then read on a Victor X5 multimode plate reader (PerkinElmer) for absorbance at 620 nm. Initial rates were determined from the slope of increasing phosphate concentration over the 15 s experimental time frame, and the Michaelis-Menten curve was fit using Prism GraphPad 10 to derive Michaelis-Menten constants (Fig. S3).

### Statistical analysis

Derived constants from individual runs generated from binding curves and Michaelis-Menten curves were evaluated for statistical significance at N = 3. To compare mutant groups to WT datasets, *p*-values were determined using Student’s unpaired *t* test and were considered statistically significant at *p* ≤ 0.05.

## Data availability

The original data for this manuscript can be obtained from the corresponding author on reasonable request.

## Supporting information

This article contains supporting information.

## Conflict of interest

The authors declare that they have no conflicts of interest with the contents of this article.
