# Supplementary material for: Motif V is an allosteric couple between the SARS-CoV-2 nsp13 nucleotide triphosphatase and helicase active sites
Source: J Biol Chem. 2026 Jan 23;302(3):111198. doi: 10.1016/j.jbc.2026.111198 (PMC12930049; doi:10.1016/j.jbc.2026.111198)
Supplement: Table S1 [file mmc1.docx]

| Supplemental Table 1. Oligonucleotides for site directed mutagenesis and helicase assays | | |
| --- | --- | --- |
| BG2033 | T532A Forward | CGACACAAGCAGTGGACTCC |
| BG2034 | T532A Reverse | GCAGACCCAAAATCTTGGACGC |
| BG2035 | S535A Forward | CGTGGACGCAAGCCAG |
| BG2036 | S535A Reverse | GTTTGTGTCGGCAGACCCAA |
| BG2291 | L405D Forward | GCCCAGGATCCGGCT |
| BG2292 | L405D Forward | CGGATCGCCAATGTAAACATAATGCT |
| BG2293 | D534A Forward | GACACAAACCGTGGCATCCAGC |
| BG2294 | D534A Reverse | GGCAGACCCAAAATCTTGGACG |
| BG1713 | AlexaFluor 488 Helicase strand | /5Alex488N/GGTAGTAATCCGCTC |
| BG1714 | Iowa Black Quencher Helicase strand | TTTTTTTTTTTTTTTTTTTTGAGCGGATTACTACC/3IABkFQ/ |
| BG1715 | Helicase competitor strand | GAGCGGATTACTACC |
| BG2261 | Unlabeled Helicase strand | GGTAGTAATCCGCTC |
| BG2262 | Unlabeled Helicase strand | TTTTTTTTTTTTTTTTTTTTGAGCGGATTACTACC |
