# Supplementary material for: Motif V is an allosteric couple between the SARS-CoV-2 nsp13 nucleotide triphosphatase and helicase active sites
Source: J Biol Chem. 2026 Jan 23;302(3):111198. doi: 10.1016/j.jbc.2026.111198 (PMC12930049; doi:10.1016/j.jbc.2026.111198)
Supplement: Supplementary Figure legends [file mmc2.docx]

**Supplemental Figure 1.** Purification and characterization of nsp13. **A)** UV trace of bacterial lysate run over a 5 mL HisTrap HP nickel column. **B)** UV trace of nickel purified lysate run over a HiLoad 16/600 Superose 6 pg size exclusion column. **C)** Coomassie stain of 12% SDS-PAGE gel of nickel column and size exclusion column fractions. **D)** Thermal shift assay of purified WT nsp13 and mutants.

**Supplemental Figure 2.** Representative raw helicase data for Michaelis-Menten data acquisition with **A** DNA varied and **B** ATP varied. Reactions were incubated in a fluorometer for 20 seconds prior to initiation with ATP and unwinding was monitored as an increase of fluorescence (left). The linear range was found during the first 5 or 10 seconds after ATP initiation, and the slopes were used to determine initial enzyme rates (middle). Rates normalized to enzyme concentration were plotted vs substrate concentration to determine Michaelis Menten data (right).

**Supplemental Figure 3.** Representative raw ATPase data for Michaelis-Menten data acquisition. Reactions were initiated with different concentrations of ATP and quenched in Biomol Green, a malachite-green based solution that colorimetrically determines phosphate concentration. A standard curve was used to quantify phosphate at each time point from absorbance and rates were calculated from slope (left). Rates normalized to enzyme concentration were plotted vs substrate concentration to determine Michaelis Menten data (right).

**Supplemental Figure 4.** ATPase assay of WT nsp13 in the presence (black circles) and absence (red squares) of 200 nM DNA indicates ATPase activity depends on bound DNA. The inactive ATPase resulted in nearly flat rate curves that fit slightly negative linear trendlines. Negative rates are a result of phosphate values being close to noise values.
