# Supplementary figures and images for: Motif V is an allosteric couple between the SARS-CoV-2 nsp13 nucleotide triphosphatase and helicase active sites
Source: J Biol Chem. 2026 Jan 23;302(3):111198. doi: 10.1016/j.jbc.2026.111198 (PMC12930049; doi:10.1016/j.jbc.2026.111198)

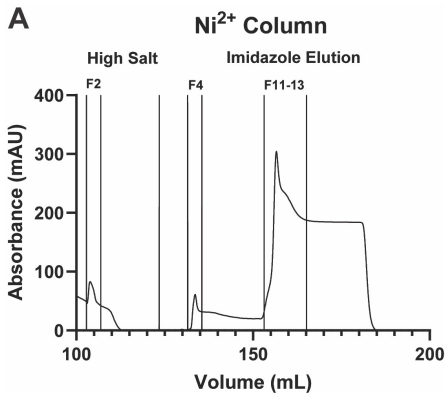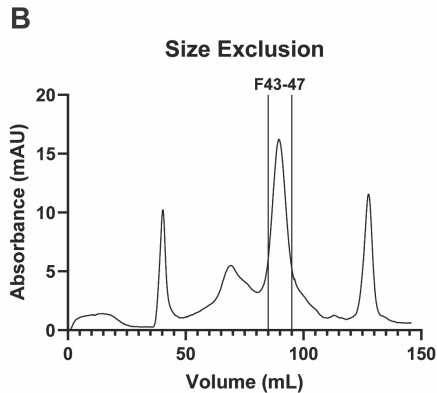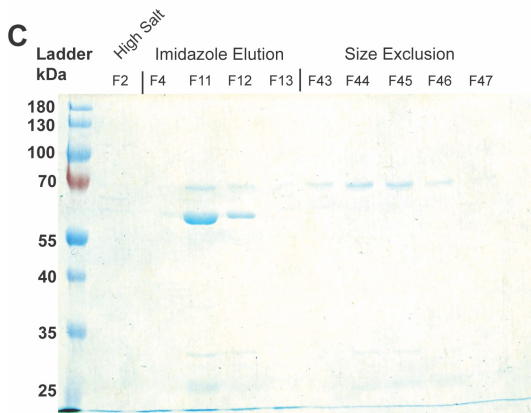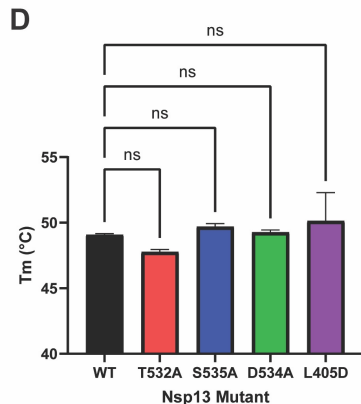

Supplement: Figure S1 [file mmc3.pdf]

### ATPase Initial Rate

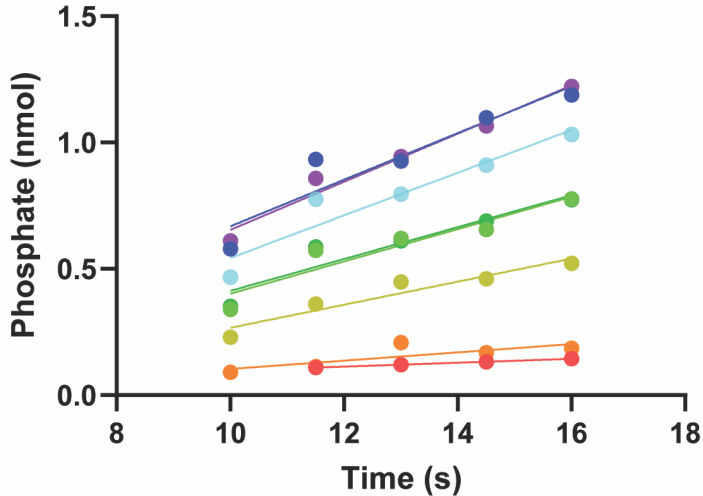

### Michaelis Menten

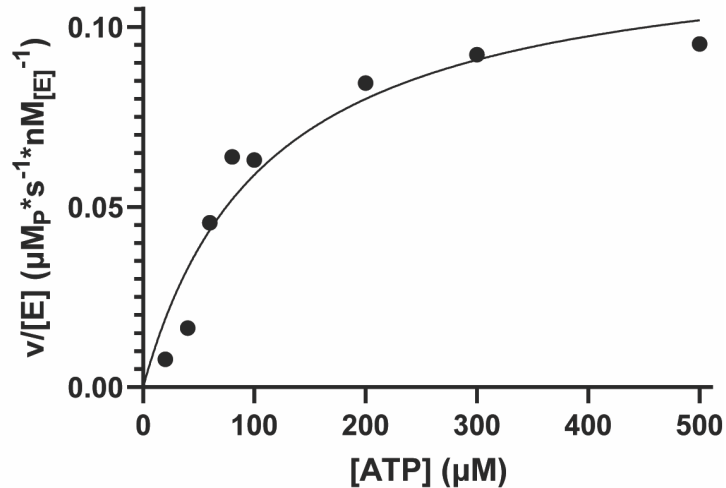

Supplement: Figure S3 [file mmc5.pdf]

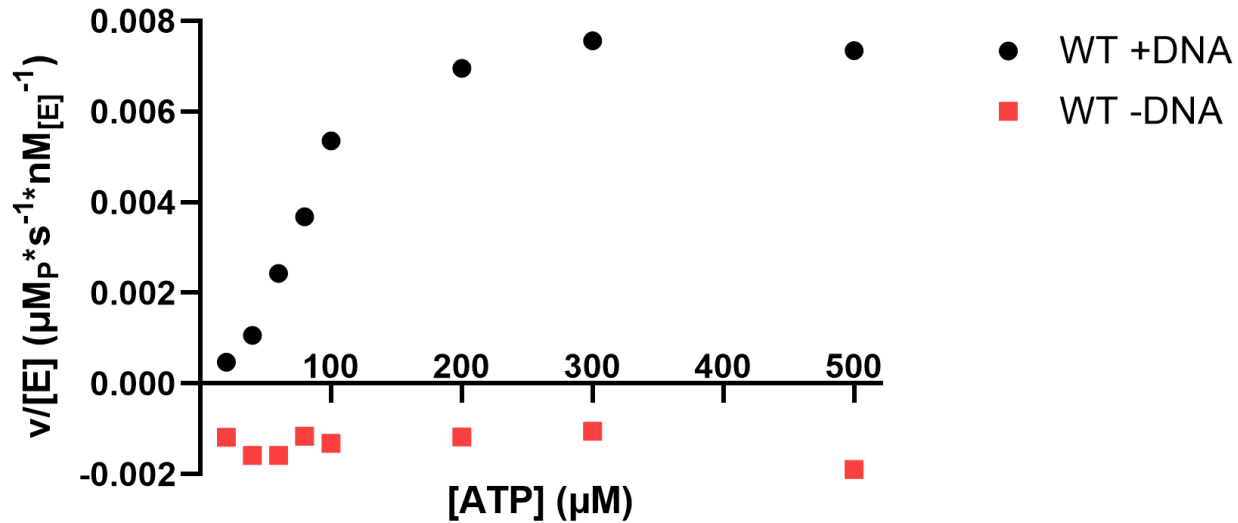

Supplement: Figure S4 [file mmc6.pdf]
