# Supplementary material for: Motif V is an allosteric couple between the SARS-CoV-2 nsp13 nucleotide triphosphatase and helicase active sites
Source: J Biol Chem. 2026 Jan 23;302(3):111198. doi: 10.1016/j.jbc.2026.111198 (PMC12930049; doi:10.1016/j.jbc.2026.111198)
Supplement: Figure S2 [file mmc4.pdf]

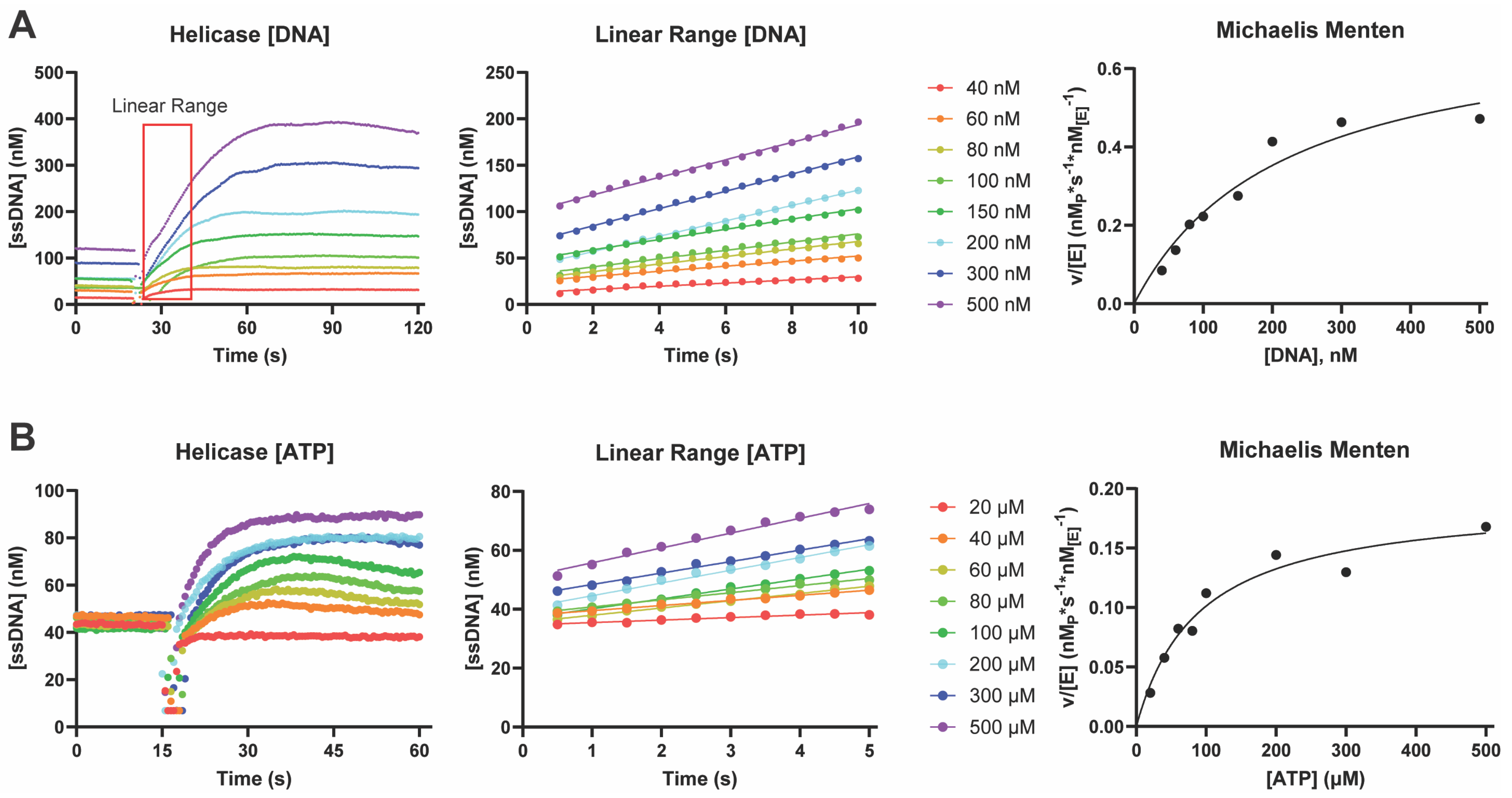
A
Helicase [DNA]
Linear Range
[ssDNA] (nM)
Time (s)
Linear Range [DNA]
[ssDNA] (nM)
Time (s)
40 nM
60 nM
80 nM
100 nM
150 nM
200 nM
300 nM
500 nM
Michaelis Menten
$v/[E]\ (nM_P{*}s^{-1}{*}nM_{[E]}^{-1})$
[DNA], nM
B
Helicase [ATP]
[ssDNA] (nM)
Time (s)
Linear Range [ATP]
[ssDNA] (nM)
Time (s)
20 μM
40 μM
60 μM
80 μM
100 μM
200 μM
300 μM
500 μM
Michaelis Menten
$v/[E]\ (nM_P{*}s^{-1}{*}nM_{[E]}^{-1})$
[ATP] (μM)
